# Mentholation affects the cigarette microbiota by selecting for bacteria resistant to harsh environmental conditions and selecting against potential bacterial pathogens

**DOI:** 10.1186/s40168-017-0235-0

**Published:** 2017-02-15

**Authors:** Jessica Chopyk, Suhana Chattopadhyay, Prachi Kulkarni, Emma Claye, Kelsey R. Babik, Molly C. Reid, Eoghan M. Smyth, Lauren E. Hittle, Joseph N. Paulson, Raul Cruz-Cano, Mihai Pop, Stephanie S. Buehler, Pamela I. Clark, Amy R. Sapkota, Emmanuel F. Mongodin

**Affiliations:** 1 Maryland Institute for Applied Environmental Health, University of Maryland School of Public Health, College Park, MD USA; 2 School of Medicine, Institute for Genome Sciences and Department of Microbiology and Immunology, University of Maryland, 801 West Baltimore Street, Office #622, Baltimore, MD 21201 USA; 3 Center for Bioinformatics and Computational Biology, University of Maryland, College Park, MD USA; 4 Department of Epidemiology and Biostatistics, University of Maryland School of Public Health, College Park, MD USA; 5 Public Health Center for Tobacco Research, Battelle, Columbus, OH USA; 6 Department of Behavioral and Community Health, University of Maryland School of Public Health, College Park, MD USA

**Keywords:** Microbiota, Cigarette, Tobacco, 16S rRNA, Pathogens, Menthol

## Abstract

**Background:**

There is a paucity of data regarding the microbial constituents of tobacco products and their impacts on public health. Moreover, there has been no comparative characterization performed on the bacterial microbiota associated with the addition of menthol, an additive that has been used by tobacco manufacturers for nearly a century. To address this knowledge gap, we conducted bacterial community profiling on tobacco from user- and custom-mentholated/non-mentholated cigarette pairs, as well as a commercially-mentholated product. Total genomic DNA was extracted using a multi-step enzymatic and mechanical lysis protocol followed by PCR amplification of the V3-V4 hypervariable regions of the 16S rRNA gene from five cigarette products (18 cigarettes per product for a total of 90 samples): Camel Crush, user-mentholated Camel Crush, Camel Kings, custom-mentholated Camel Kings, and Newport Menthols. Sequencing was performed on the Illumina MiSeq platform and sequences were processed using the Quantitative Insights Into Microbial Ecology (QIIME) software package.

**Results:**

In all products, *Pseudomonas* was the most abundant genera and included *Pseudomonas oryzihabitans* and *Pseudomonas putida,* regardless of mentholation status. However, further comparative analysis of the five products revealed significant differences in the bacterial compositions across products. Bacterial community richness was higher among non-mentholated products compared to those that were mentholated, particularly those that were custom-mentholated. In addition, mentholation appeared to be correlated with a reduction in potential human bacterial pathogens and an increase in bacterial species resistant to harsh environmental conditions.

**Conclusions:**

Taken together, these data provide preliminary evidence that the mentholation of commercially available cigarettes can impact the bacterial community of these products.

**Electronic supplementary material:**

The online version of this article (doi:10.1186/s40168-017-0235-0) contains supplementary material, which is available to authorized users.

## Background

In 2014, an estimated 264 billion cigarettes were sold in the USA, about one-quarter of which were mentholated products [1, 2]. Menthol, a cyclic terpene alcohol, is known to activate cold receptors and provide a “cooling” sensation [3, 4]. In the 1920s, cigarette companies began using this additive to reduce the harshness of cigarette products and to appeal to a wider spectrum of consumers [5, 6]. Although non-menthol cigarettes do contain low levels of menthol, levels in cigarette products labeled as mentholated are 50–5000 times higher [7]. For commercially produced menthol cigarettes, menthol, which is usually plant-derived or produced synthetically, is added directly to the tobacco or to other parts of the cigarette (e.g., filter, filter paper) [8]. In addition, several brands of cigarettes (e.g., Camel Crush) have capsules embedded in the filter, which can be “crushed” by the user to release a menthol-containing solution. Today, young adults, minority groups, adult women, and members of low-income households are the primary consumers of menthol cigarettes [2, 9, 10].

Previous studies have provided evidence that menthol smokers are characterized by decreased nicotine metabolism, enhanced systemic nicotine exposure [11], increased serum cotinine levels [12], and increased levels of carboxyhemoglobin [12, 13]. The presence of menthol in some cigarette products has also been shown to increase levels of volatile organic compounds in mainstream smoke [14] and inhibit the detoxification of carcinogens in liver microsome studies [15]. Although results are mixed [16, 17], it appears that menthol cigarettes may be more addictive and may convey a greater risk of cancer and other tobacco-related diseases compared to non-mentholated cigarettes [18, 19]. However, there are relatively few studies that have evaluated other physiological and toxicological health effects associated with exposure to menthol cigarettes, including the impact of the bacteria associated with these products on smokers’ oral health.

The antibacterial nature of menthol has been shown to inhibit human and plant pathogenic microorganisms; however, its reaction with the bacterial constituents of the cigarette microenvironment has yet to be explored [20]. The history of microorganisms in tobacco has been documented by several groups [21], with researchers as early as the late 1890s beginning to characterize the microbiology of tobacco before and during fermentation. Fast-forwarding to the 1950s and 1960s, major tobacco companies and researchers began to produce reports describing total numbers of cultivable bacteria in tobacco products [21–24]. More recently, several groups have used traditional, culture-dependent methods to identify and characterize specific bacterial and fungal species present in tobacco products including *Actinomycetes* spp. [25], *Pantoea* spp. [26], *Kurthia* spp. [27], *Bacillus* spp. [27], and *Mycobacterium avium* (an important respiratory pathogen) [28].

One study, in particular, recovered viable *M. avium* from cigarette tobacco, tobacco paper and the cigarette filters before cigarettes were smoked and subsequently recovered viable *M. avium* from the cigarette filters after the cigarettes were smoked [28]. These data provide evidence that *M. avium* can survive exposures to high temperatures and gases generated during the cigarette combustion process and potentially be inhaled in mainstream smoke [28]. Other studies have shown that the mainstream smoke of combustible tobacco products also contains other microbial constituents, including lipopolysaccharides, peptidoglycan fragments and fungal components [26]. The same study also showed that cigarettes kept at 94% relative humidity for over 8 days were characterized by additional bacterial and fungal growth within the cigarette tobacco, further demonstrating that microorganisms present in the tobacco are viable and metabolically active [26]. Moreover, in a study by Pauly et al. [24], bacteria growing on single tobacco flakes from multiple cigarette brands were characterized, and the authors hypothesized that these tobacco-associated microorganisms could represent a health risk to the smoker as they are carried to the lungs on the surface of tobacco particulate matter generated during smoking. The impact of these microbial exposures on tobacco users’ health is still unclear, as very few epidemiologic studies have focused on the public health impacts associated with the microbiological components of tobacco products. However, bacteria in cigarettes have been previously associated with acute eosinophilic pneumonitis in military personnel deployed in operation Iraqi Freedom, emphasizing the critical role that these microorganisms might play in acute and chronic conditions among tobacco users [27].

Culture-based methods that are used to assess the microbiology of cigarettes, as well as the impacts of menthol on bacterial populations, are limited due to the small percentage of bacterial species that can be cultured in the laboratory. Previous work by our group aimed to address this knowledge gap by applying a 16S rRNA gene-based taxonomic microarray approach to evaluate total bacterial diversity of commercially available cigarettes [29]. In all tested products, 15 different classes of bacteria and a broad array of potentially pathogenic microorganisms were identified, including *Acinetobacter* spp., *Bacillus* spp., *Clostridium* spp., *Klebsiella* spp., *Pseudomonas aeruginosa* spp., and *Serratia* spp. [29]. This initial study also provided some preliminary evidence that the bacterial microbiota of menthol vs. non-menthol cigarettes may vary. However, due to the relatively small number of bacterial taxa represented on the microarray used in the previous study, our view of the bacterial diversity within the tested products was limited.

Therefore, in this study, we applied high-throughput next generation sequencing—which provides a much broader view of total bacterial diversity—to characterize five cigarette products: Camel Crush, user-mentholated Camel Crush, Camel Kings, custom-mentholated Camel Kings, and Newport Menthols. In addition to comparing mentholated and non-mentholated cigarette pairs we aimed to identify potential bacterial pathogens that users may be exposed to when they smoke these products, and expand our understanding of the scope of bacterial diversity present in mentholated and non-mentholated cigarette tobacco.

## Methods

### Sample collection

In the Spring of 2014, menthol and non-menthol cigarettes were either purchased from selected tobacco stores in College Park, Maryland or provided by our collaborators at The Battelle Public Health Center for Tobacco Research (Columbus, OH) (Table 1). The following products were purchased from selected tobacco stores in College Park, MD: (1) Camel Crush, regular, fresh (CC) (Camel Crush; R.J. Reynolds Tobacco Co., Winston-Salem, NC, USA), where the capsule within the filter was subsequently not crushed during the study; (2) Camel Crush, regular, fresh (CCM), where the capsule was subsequently crushed during the study to release a menthol-containing solution into the cigarette filter (user mentholated) (CCM); and (3) a commercially mentholated brand, Newport Menthol Box (NMB) (Lorillard Tobacco Co., Greensboro, NC, USA). The following products were provide by Battelle: 4) Camel full flavor, hard pack, king (CK) (Camel Kings; R.J. Reynolds Tobacco Co., Winston-Salem, NC, USA); and 5) Camel Kings that were custom-mentholated by Battelle (CKM) using a vapor deposition technique described in detail in MacGregor et al. [30]. The custom-mentholated Camel Kings were prepared concurrently in three separate chambers [30]. The Camel Kings that were not mentholated went through the same motions and preparations and were handled in the same exact way as those that were mentholated. The only difference was that the non-mentholated Camel Kings were not exposed to the mentholation chamber. All custom-mentholated and non-mentholated Camel Kings were shipped from Battelle on the day that custom-mentholation was completed via overnight carrier without refrigeration and all cigarettes were subsequently stored at room temperature until processing. We included two pairs of mentholated and non-mentholated products (custom-mentholated Camel Kings versus non-mentholated Camel Kings; and “non-crushed” Camel Crush cigarettes versus “crushed” Camel Crush cigarettes, as described above) so that we could specifically evaluate the influence of the addition of menthol into two different products on the bacterial community composition of those products. Three lots of each cigarette product were tested in replicates of 6 for a total of 90 samples (18 cigarettes per brand) tested during the study.

**Table 1:** Descriptions of cigarette products tested

Cigarette product	Menthol status	Abbreviation
Camel King filters	Non-menthol	CK
Camel King filters	Mentholated (custom)^a^	CKM
Camel Crush	Non-menthol^b^	CC
Camel Crush	Mentholated (user)^c^	CCM
Newport Menthol Box	Mentholated (manufacturer)^d^	NMB

^a^Mentholated at The Battelle Public Health Center for Tobacco Research

^b^Camel Crush capsule within the filter was not crushed

^c^Camel Crush capsule within the filter was crushed in the laboratory prior to DNA extraction

^d^Commercially mentholated by the manufacturer

### DNA extraction

DNA extraction was performed on cigarettes from freshly opened packages, with the exception of the custom-mentholated and non-mentholated Camel Kings (CK and CKM), which were opened at Battelle, processed and shipped as described above. Our total DNA extraction protocol was adapted from procedures previously published [31, 32]. Briefly, each cigarette was dissected under sterile conditions, and 0.2 g of tobacco was weighed out and aseptically placed in Lysing Matrix B tubes (MP Biomedicals, Solon, OH). Enzymatic lysis was initiated by adding the following to the tubes containing cigarette tobacco and lysing matrix: 1 ml of ice cold 1 × molecular grade PBS buffer (Gibco by Life Technologies, NY), 5 μl lysozyme from chicken egg white (10 mg/ml, Sigma-Aldrich, MO), 5 μl lysostaphin from *Staphylococcus staphylolyticus* (5 mg/ml, Sigma-Aldrich, MO) and 15 μl of mutanolysin from *Streptomyces globisporus* ATCC 21553 (1 mg/ml, Sigma-Aldrich, MO). Tubes were then incubated at 37 °C for 30 min, after which a second enzymatic cocktail was added to each tube, composed of 10 μl Proteinase K (20 mg/ml, Invitrogen by Life Technologies, NY) and 50 μl of SDS (10% w/v, BioRad). Following incubation at 55 °C for 45 min, the samples were then further lysed mechanically using a FastPrep Instrument FP-24 (MP Biomedicals, CA) at 6.0 m/s for 40s. The resulting lysate was centrifuged for 3 min at 10,000 rcf and DNA was purified using the QIAmp DSP DNA mini kit 50, v2 (Qiagen, CA), according to the manufacturer’s protocol. Six replicate DNA extractions were completed on each sample and negative extraction controls were included to ensure that no exogenous DNA contaminated the samples during extraction. DNA quality control/quality assurance was performed using spectrophotometric measurements on a NanoDrop™ (Thermo Scientific, City, State), as well as gel electrophoresis.

### 16S rRNA gene PCR amplification and sequencing

The V3-V4 hypervariable region of the 16S rRNA gene was PCR-amplified and sequenced on Illumina MiSeq (Illumina, San Diego, CA) using a dual-indexing strategy for multiplexed sequencing developed at the Institute for Genome Sciences and described in detail previously [33].

Briefly, PCR reactions were set-up in 96-well microtiter plates using the 319 F (ACTCCTACGGGAGGCAGCAG) and 806R (GGACTACHVGGGTWTCTAAT) universal primers, each of which also included a linker sequence required for Illumina MiSeq 300 bp paired-ends sequencing, and a 12-bp heterogeneity-spacer index sequence aimed at minimizing biases associated with low-diversity amplicons sequencing [33, 34]. This sample multiplexing approach ensured that 500 samples could be multiplexed in a single Illumina MiSeq run. PCR amplifications were performed using Phusion High- Fidelity DNA polymerase (Thermo Fisher, USA) and 2 ng of template DNA in a total reaction volume of 25 μl. Because of the presence of PCR inhibitors in the DNA solution, an additional 0.375 μl of bovine serum albumin (BSA) (20 mg/ml, Sigma) was added to the PCR reactions. Reactions were run in a DNA Engine Tetrad 2 thermo cycler (Bio-Rad, USA) using the following cycling parameters: 30 s at 98 °C, followed by 30 cycles of 10 s at 98 °C, 15 s at 66 °C, and 15 s at 72 °C, with a final step of 10 min at 72 °C. Negative controls without DNA template were performed for each primer pair. The presence of amplicons was confirmed using gel electrophoresis, after which the SequalPrep Normalization Plate kit (Invitrogen Inc., CA, USA) was used for clean-up and normalization (25 ng of 16S PCR amplicons from each sample were included), before pooling and 16S rRNA sequencing using the Illumina MiSeq (Illumina, San Diego, CA) according to the manufacturer’s protocol.

### Sequence quality filtering and analysis of 16S rRNA gene sequences

16S rRNA reads were initially screened for low quality bases and short read lengths [33]. Paired-end read pairs were then assembled using PANDAseq [35] and the resulting consensus sequences were de-multiplexed (i.e., assigned to their original sample), trimmed of artificial barcodes and primers, and assessed for chimeras using UCHIME in de novo mode implemented in Quantitative Insights Into Microbial Ecology (QIIME ; release v. 1.9) [36]. Quality trimmed sequences were then clustered de novo into Operational Taxonomic Units (OTUs) with the SILVA 16S database [37] in QIIME [36], with a minimum confidence threshold of 0.97 for the taxonomic assignments. All sequences taxonomically assigned to chloroplasts were removed from further downstream analysis. Data were normalized to account for uneven sampling depth with metagenomeSeq’s cumulative sum scaling [38], a novel normalization method that has been shown to be less biased than the standard approach (total sum normalization).

Taxonomic assignments of the most abundant genera, contributing >1% of the total abundance in at least one sample, were obtained through QIIME [36] and visualized with RStudio Version 0.99.473 and vegan [39], gplots [40], RColorBrewer [41] and heatplus [42] R packages. Prior to normalization, alpha diversity was estimated with the Chao1 estimator [43], and the Shannon Index [44] through the R packages: Bioconductor [45], metagenomeSeq [46], vegan [39] phyloseq [47] and fossil [48]. To account for uneven sampling depth, the data were also rarefied to the minimum sampling depth of 631 sequences. Alpha diversity data was tested for significance using a Tukey test. Beta diversity was determined through principle coordinates analysis (PCoA) plots of Bray-Curtis distance performed through QIIME and tested for significance with ANOSIM (9,999 permutations) [49].

Determination of statistically significant differences (*p* < 0.05) in OTU bacterial relative abundance between mentholated cigarette products and their non-mentholated counterpart (mentholated Camel Crush vs. non-mentholated Camel Crush and custom-mentholated Camel Kings vs. non-mentholated Camel Kings) was performed using DESeq2 [50] through QIIME [36], which utilizes Benjamini-Hochberg multiple-inference correction. DESeq was used due to its high power in computing smaller sample sizes (<20 samples per group) [51]. The significant OTUs (*p* < 0.05) were visualized with RStudio Version 0.99.473 and R packages ggplot2 [52], vegan [39], and phyloseq [47]. In addition, species-level assignments were performed for OTUs of interest: reference sequences matching assigned genera of each OTU were extracted from the Ribosomal Database Project (RDP; http://rdp.cme.msu.edu/), aligned with the sequences from the OTU(s) of interest via MAFFT [53], and the V3-V4 region extracted. An unrooted maximum likelihood tree with 10 bootstrap replicates was generated with PhyML [54] for each of the alignments. Trees were visualized with FigTree [55] and branches colored based on species.

## Results

### Sequencing data and taxonomic assignments

All 90 cigarette samples were successfully PCR amplified and sequenced, thus validating our DNA extraction and purification protocol. A total of 2046 different bacterial OTUs (97% identity) were identified from a total of 909,053 sequences across all samples, and the average number of sequences per sample was 10,100 (+/− 5004 SD, Additional file 1: Figure S1).

**Fig. 1:**
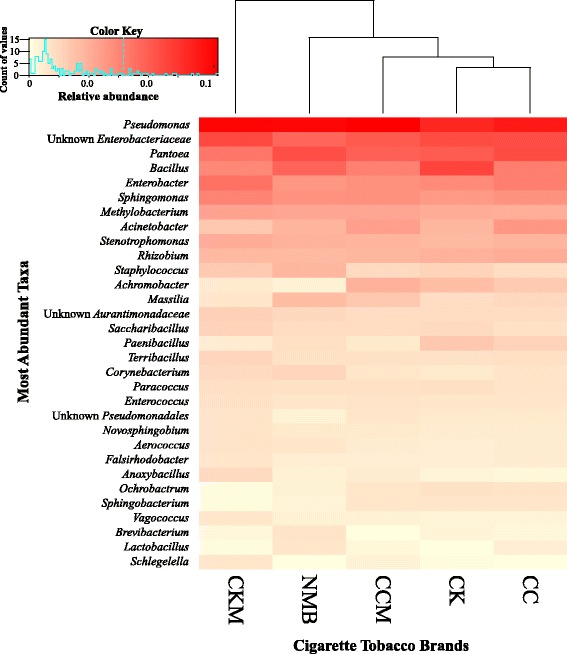
Heat map showing the relative abundances of the most dominant bacterial genera identified (>1%) in tested cigarette products. Samples pooled by product type: Camel Crush (CC), mentholated Camel Crush (CCM), Camel Kings (CK), custom-mentholated Camel Kings (CKM), and Newport Menthol Box (NMB). Hierarchical clustering of the pooled samples is represented by the dendrogram at the *top* and *inside the color key* shows a histogram of the count of the individual values

The average relative abundance of the most dominant genera (>1.0% in at least one sample) showed that, across all brands, bacteria from the genus *Pseudomonas* dominated, followed by unclassified members of the *Enterobacteriaceae* family, and members of the *Pantoea* and *Bacillus* genera (Fig. 1). Members from the *Pseudomonas* genus were comprised of 15 unique OTUs, with 7 *Pseudomonas* OTUs shared between all mentholation states (OTU#s 1532, 10, 134, 1868, 1886, 8, and 3). Some of these shared *Pseudomonas* OTUs were assigned via RDP classification using SILVA to *Pseudomonas oryzihabitans* (OTUs #1868 and 6) and *Pseudomonas putida* (OTU #3) species. Certain OTUs were also unique to the different menthol products, including OTU #1250 and OTU #1137 for NMB and OTU #77 for CCM. Species level taxonomic information was assigned only to OTU #1137, *Pseudomonas fulva* species. In addition, heatmap hierarchical clustering of the samples revealed that the bacterial community profiles were more similar between the non-menthol cigarette products CK and CC, compared to the commercially mentholated (NMB) and custom-mentholated (CKM) products (Fig. 1).

### Alpha and beta diversity metrics by product and menthol state

**Fig. 2:**
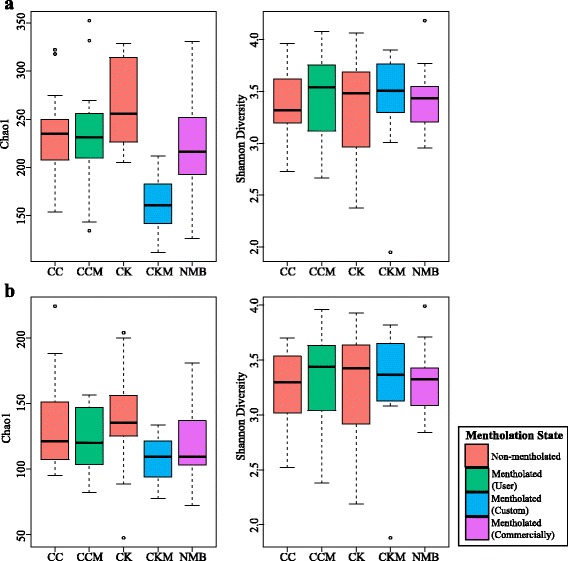
Box plots showing alpha diversity (Chao1 richness estimator and Shannon Index) variation across samples on non-rarefied data (**a**) and with data rarefied to the minimum sampling depth (**b**). *Bars* are colored by mentholation status: *red bars*—non-mentholated; *green bars*—user mentholated; *blue bars*—custom-mentholated; *purple bars*—commercially-mentholated

Because sequence coverage can have an impact on measuring alpha diversity, a quantification of intra-sample diversity, we employed Chao1 and Shannon indices (Fig. 2) on both non-rarefied (Fig. 2a) and rarefied data (Fig. 2b). Tobacco-associated microbiota from the custom-mentholated Camel King (CKM) exhibited significantly lower Chao1 diversity (*p* value <0.05) compared to its non-mentholated counterpart (CK), regardless of rarefaction (Fig. 2).

**Fig. 3:**
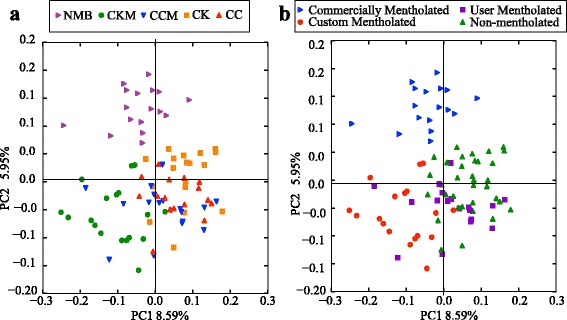
PCoA analysis plots of Bray-Curtis computed distances between cigarette products. **a** Points colored by brand: *purple*—Newport Menthol (NMB); *green*—mentholated Camel King (CKM); *blue*—mentholated Camel Crush (CCM); *orange*—Camel Kings (CK); *red*—Camel Crush (CC) (ANOSIM R value = 0.35, *p* value = 0.0001), (**b**) Points colored by mentholation status: *green*—non-mentholated; *purple*—user mentholated; *blue*—commercially-mentholated; *red*—custom mentholated. (ANOSIM *R* = 0.43, *p* = 0.0001)

To quantify inter-sample diversity (beta diversity), principal coordinate analyses using the Bray Curtis distance, a measure widely used to measure the compositional dissimilarity between two different sites in ecology and microbiome studies, were performed. Separation of the tobacco-associated bacterial profiles was evident along the first principal component (PC1), which explained 8.59% of the total variability between communities, and the second principal component (PC2), which explained 5.95% of the total variability between communities by brand (ANOSIM *R* = 0.35, *p* = 0.0001) and mentholation status (ANOSIM *R* = 0.43, *p* = 0.0001) (Fig. 3). This was especially evident for the commercially-mentholated, custom-mentholated and non-mentholated products. Unweighted and weighted UniFrac distances [56] were also used to measure beta diversity between the brands (Additional file 1: Figure S2), (ANOSIM R value = 0.25, *p* = 0.0001) and (ANOSIM *R* = 0.16, *p* = 0.0001), respectively.

### Taxonomic analysis by product and menthol status

**Fig. 4:**
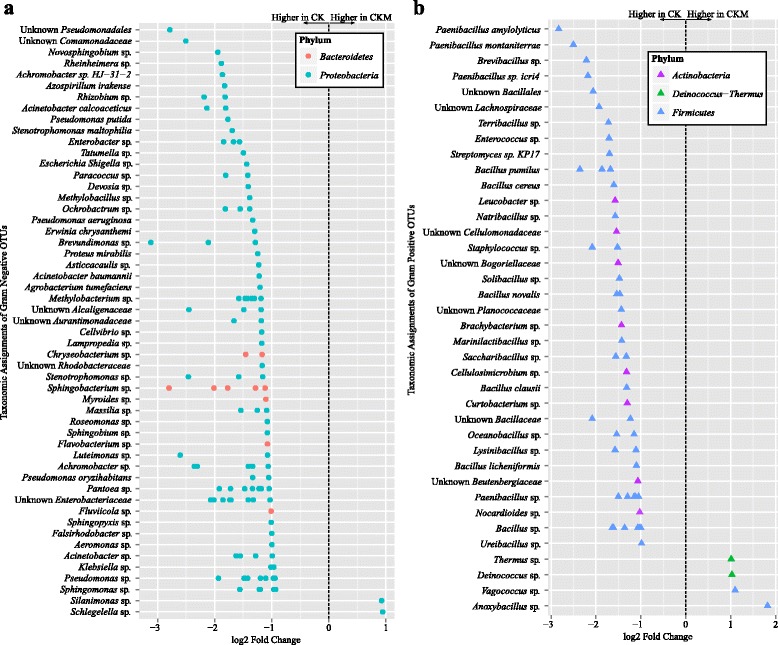
Overview of relative abundances of bacterial OTUs that were statistically significantly different (*p* < 0.05) between custom-mentholated Camel Kings (CKM) and non-mentholated Camel Kings (CK). OTUs are colored by Phylum and differentiated by Gram negative (**a**) and Gram positive (**b**) classification. A positive log2-fold change value denotes an OTU that is significantly higher in custom-mentholated Camel Kings, while a negative log2-fold change indicates an OTU that is significantly higher in non-mentholated Camel Kings. The *dotted line* and *arrows* highlight the conversion in log2-fold change from negative to positive values

There were 173 OTUs at statistically significantly (*p* < 0.05) different relative abundances between custom-mentholated Camel Kings and non-mentholated Camel Kings (Additional file 1: Table S1, Fig. 4). Out of these, 167 OTUs were at lower relative abundance in the custom-mentholated Camel Kings, of which 116 were Gram-negative (Fig. 4a), with species level assignments including *Achromobacter sp. HJ-31-2* (OTU #16)*, Azospirillum irakense* (OTU #167)*, Acinetobacter calcoaceticus* (OTU #40)*, Pseudomonas putida* (OTU #3), *Stenotrophomonas maltophilia* (OTU #15)*, Pseudomonas aeruginosa* (OTU #420), *Erwinia chrysanthemi* (OTU #446)*, Proteus mirabilis* (OTU #450), *Acinetobacter baumannii* (OTU #29), *Agrobacterium tumefaciens* (OTU #1998), and *Pseudomonas oryzihabitans* (OTU #1868). The remaining 51 OTUs at lower relative abundance in custom-mentholated Camel Kings were Gram-positive (Fig. 4b), with species level assignments including *Paenibacillus amylolyticus* (OTU #37), *Paenibacillus montaniterrae* (OTU #91), *Paenibacillus sp. icri4* (OTU #51), *Streptomyces sp. KP17* (OTU #52), *Bacillus pumilus* (OTU #1937, 5, 1948), *Bacillus cereus* (OTU #176), *Bacillus novalis* (OTU #530 and 1442), *Bacillus clausii* (OTU #9), and *Bacillus licheniformis* (OTU #41). In addition, six OTUs were at higher relative abundance in the custom-mentholated Camel Kings and were composed of two Gram-negative bacteria, *Schlegelella* sp. (OTU #87) and *Silanimonas* sp. (OTU #207) (Fig. 4a), and four Gram-positive bacteria, *Anoxybacillus* sp. (OTU #31), *Vagococcus* sp. (OTU #54), *Deinococcus* sp. (OTU #272), and *Thermus* sp. (OTU #266) (Fig. 4b).

**Fig. 5:**
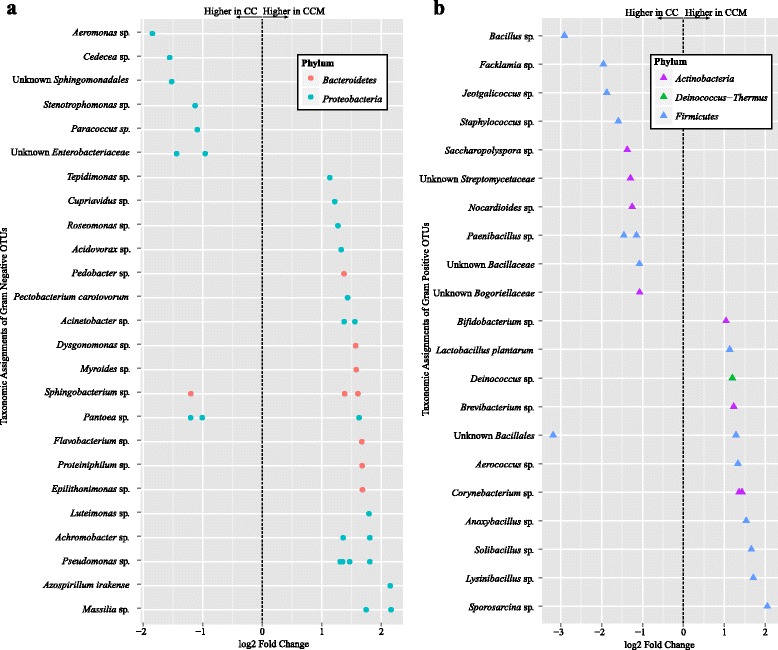
Overview of relative abundances of OTUs that were statistically significantly different (*p* < 0.05) between mentholated Camel Crush (CCM) and non-mentholated Camel Crush (CC). OTUs are colored by Phylum and differentiated by Gram negative (**a**) and Gram positive (**b**) classification. A positive log2-fold change value denotes an OTU that is significantly higher in mentholated Camel Crush, while a negative log2-fold change indicates an OTU that is significantly higher in non-mentholated Camel Crush. The *dotted line* and *arrows* highlight the conversion in log2-fold change from negative to positive values

There were 60 OTUs at statistically significantly (*p* < 0.05) different relative abundances between mentholated Camel Crush and non-mentholated Camel Crush (Additional file 1: Table S2, Fig. 5). Twenty-two OTUs were at lower relative abundance in the mentholated Camel Crush and, of these, 10 were Gram-negative OTUs (Fig. 5a) including *Aeromonas* sp. (OTU #285), *Cedecea* sp. (OTU #783), unknown *Sphingomonadales* (OTU #333), *Stenotrophomonas* sp. (OTU #1682), *Paracoccus* sp. (OTU #289), unknown *Enterobacteriaceae* (OTU #1969, 2017), *Sphingobacterium* sp. (OTU #124), and *Pantoea* sp. (OTU #398 and 1448). The remaining 12 OTUs at lower relative abundance in mentholated Camel Crush were Gram-positive (Fig. 5b) and included *Bacillus* sp. (OTU #30), *Facklamia* sp. (OTU #104), *Jeotgalicoccus* sp. (OTU #73), *Staphylococcus* sp. (OTU #143), *Saccharopolyspora* sp. (OTU #293), unknown *Streptomycetaceae* (OTU #1729), *Nocardioides* sp. (OTU #86), *Paenibacillus* sp. (OTU #128 and 340), unknown *Bacillaceae* (OTU #296), unknown *Bogoriellaceae* (OTU #193), and unknown *Bacillales* (OTU #667). Additionally, 38 OTUs were at higher relative abundance in the mentholated Camel Crush samples and consisted of 26 Gram-negative OTUs, with species assignments for *Azospirillum irakense* (OTU #167) and *Pectobacterium carotovorum* (OTU #48). The remaining 12 were Gram-positive and included *Sporosarcina* sp*.* (OTU #228), *Lysinibacillus* sp*.* (OTU #93), *Solibacillus* sp*.* (OTU #90), *Anoxybacillus* sp*.* (OTU #31), *Corynebacterium* sp*.* (OTU #21 and 551), *Aerococcus* sp*.* (OTU #14), unknown *Bacillales* (OTU #1182), *Brevibacterium* sp*.* (OTU #153), *Deinococcus* sp*.* (OTU #272), *Lactobacillus plantarum* (OTU #359), and *Bifidobacterium* sp*.* (OTU #535).

OTUs of interest were selected to confirm or predict species-level assignments via phylogenic analyses (Additional file 1: Figures S3 to S10). OTUs included *Pseudomonas putida* (OTU #3), *Pseudomonas oryzihabitans* (OTU #8, 1868), *Pseudomonas* sp. (OTU #10, 77, 132, 134, 163, 251, 608, 972, 1250, 1532, 1872, 1886), *Pseudomonas aeruginosa* (OTU #420), *Pseudomonas fulva* (OTU #1137), *Actinobacter* sp. (OTU #12, 182, 247, 870, 1900), *Acinetobacter baumannii* (OTU #29), *Acinetobacter calcoaceticus* (OTU #40, 496), *Proteus mirabilis* (OTU #450),

*Anoxybacillus* sp. (OTU #31), *Vagococcus* sp. (OTU #54), *Deinococcus* sp. (OTU #272), *Thermus* sp. (OTU #266), *Stenotrophomonas* sp. (OTU #1682, 1899, 1913), and *Stenotrophomonas maltophilia* (OTU #15).

Distinct phylogenetic clustering could be seen among the OTUs and representative species (Additional file 1: Figures S3-S10). *Pseudomonas oryzihabitans* OTU #8 and 1868 and *Pseudomonas aeruginosa* OTU #420 claded with strains of their assigned species. *Pseudomonas* sp. OTU #10 and 1886 grouped closely with strains of *Pseudomonas putida*, while OTUs #251, 1250, and 134 claded with strains of *Pseudomonas stutzeri* (Additional file 1: Figure S3). Although close to several strains of *Pseudomonas putida*, OTU #3 did not group within the large clades of this species (Additional file 1: Figure S3).

*Acinetobacter baumannii* (OTU #29) and *Acinetobacter calcoaceticus* (OTU #496) also clustered with strains of their assigned species (Additional file 1: Figure S4). Additionally, *Actinobacter* sp. OTU 12 grouped with *Acinetobacter baumannii* (Additional file 1: Figure S4). *Stenotrophomonas sp.* OTUs #1913 and 1682 appeared close to one another within a clade of *Stenotrophomonas maltophilia. Stenotrophomonas maltophilia* OTU #15 claded further away from OTUs #1913 and 1682*,* but was also with strains of *Stenotrophomonas maltophilia* (Additional file 1: Figure S5). *Stenotrophomonas* sp. OTU #1899 appeared most phylogenetically related to strains of *Stenotrophomonas chelatiphaga* (Additional file 1: Figure S5). *Anoxybacillus* sp. (OTU #31) claded closely to strains of *Anoxybacillus flavithermus, Deinococcus* sp. (OTU #272) appeared closely related to strains of *Deinococcus geothermalis,* and *Thermus* sp. (OTU #266) claded closely to *Thermus scotoductus* (Additional file 1: Figure S6-8)*.*

## Discussion

It has been well established that smokers and those exposed to secondhand smoke are more susceptible to bacterial infections than are non-smokers [57]. Therefore, characterizing this exposure and, more specifically, the bacterial components of cigarette tobacco and their additives, is an important step in uncovering the relationship between tobacco products and user-health. This study aimed to provide comprehensive data concerning bacterial communities present in mentholated and non-mentholated cigarettes by utilizing next-generation sequencing technologies that, to date, have been underutilized in the field of tobacco regulatory science.

The most abundant genus detected in all cigarette products tested, regardless of mentholation status, was *Pseudomonas* (Fig. 1)*.* This was not unexpected as species of *Pseudomonas* are ubiquitous in aquatic and terrestrial environments and have been hypothesized to be a part of the core pulmonary bacterial microbiome [58]. *Pseudomonas* spp*.* have also been implicated as the dominant genus in cases of chronic obstructive pulmonary disease (COPD) [58], cystic fibrosis [59], and subjects with decreased lung function [58], making their high prevalence and high abundance within cigarette tobacco a potential human health concern. *Pseudomonas putida* due to its metabolical versatility has a distinct association with tobacco and human disease [60]. For instance, several strains of *Pseudomonas putida* (e.g. S16, J5, SKD, and ZB-16A) have the ability to degrade nicotine [61–65], while others have emerged as significant human pathogens causing urinary tract infections [66, 67] and nosocomial pneumonia [66, 68], particularly in ill or immunocompromised patients. In addition, it has been suggested that the clinical isolate strain, HB3267, acquired antibiotic and biocide resistance genes from opportunistic human pathogens, including *Acinetobacter baumannii* [69], which was one of the species found at higher relative abundance in Camel Kings compared to its mentholated counterpart (Fig. 4). *Acinetobacter baumannii* is a Gram-negative opportunistic pathogen of particular global concern due to its increasing rates of antibiotic resistance [70–72] and connection to nosocomial pneumonia and ventilator-associated pneumonia in patients with underlying lung disease [70, 73, 74].

Additional common and rare bacterial species—some of which are known to cause respiratory illnesses—were found at higher relative abundances in the non-mentholated Camel Kings compared to the custom-mentholated Camel Kings, including *Pseudomonas oryzihabitans* and *Pseudomonas aeruginosa. Pseudomonas aeruginosa* is noteworthy as a member of the tobacco microenvironment not only due to its association with the occurrence and exacerbation of COPD but also due to its response to cigarette smoke [75–77]. A study preformed on murine models showed that exposure to cigarette smoke followed by infection with *Pseudomonas aeruginosa* resulted in delayed clearance of infection and increased morbidity compared to controls [77].

Despite the overall decrease in bacterial diversity and potential human pathogens that we observed in custom-mentholated compared to non-mentholated Camel Kings, we detected statistically significant (*p* < 0.05) increases in the relative abundance of four Gram-positive bacterial species (*Thermus* sp., *Deinococcus* sp., *Vagococcus* sp., and *Anoxybacillus* sp.) and two Gram-negative species (*Silanimonas* sp. and *Schlegelella* sp.) in the mentholated product. Interestingly, *Anoxybacillus* and *Deinococcus* include species that are able to withstand extreme environmental conditions (e.g., elevated pH, industrial processes, UV treatment, radiation) [78–81], possibly due to the production of protective carotenoids found in strains of both genera [82–84]. Furthermore, species of *Thermus* [85], *Silanimonas* [86] and *Schlegelella* [75] are known to be thermophilic, hyperthermophilic and/or alkaliphilic. For example, strains of *Thermus scotoductus* have been isolated from a hot water pipeline [87], a South African gold mine [88], and a sulfide-rich neutral hot spring [89].

These data suggest that menthol may be effective against Gram-negative bacteria in cigarette products and select and/or introduce resilient bacterial species that can tolerate the antibacterial activity of menthol. Menthol, although known to be active against both Gram-positive and Gram-negative bacteria [20], has shown, in some instances, to be more effective against Gram-negative bacteria, especially compared to other essential oils [90]. Nevertheless, this overall trend was not observed in our comparisons between the user-mentholated Camel Crush and the non-mentholated Camel Crush (Fig. 5). This finding may be due to the degree and rate of menthol exposure in the user-mentholated Camel Crush products. Because these cigarettes are user-mentholated (by crushing a capsule within the cigarette filter and releasing a menthol-containing solution immediately before use), the tobacco is generally exposed to the antibacterial effects of menthol only for a brief period of time before consumption, if at all. For these products, we only evaluated a single time point, just following menthol release; as a result the menthol may not have had the opportunity to migrate fully to the tobacco.

Our study had other limitations as well. We detected more than 2000 OTUs, but as with all DNA-based 16S rRNA gene-sequencing studies, future studies are required to confirm whether these bacteria are active and capable of potentially colonizing a user exposed to these microorganisms. It is also important to note that, chemically, the only difference between the tobacco content of the custom-mentholated Camel King and non-mentholated Camel King cigarettes was the addition of L-menthol. However, the mentholation process used to produce the custom-mentholated Camel King could not be performed under DNA-free conditions, and the introduction of low levels of contaminating foreign bacterial DNA, although unlikely, could be a possibility. Furthermore, commercially available cigarettes may differ from each other in more ways than menthol content, such as tobacco blend [8]. However, the presence of increasing *Anoxybacillus* and *Deinococcus* OTUs in both custom and user-mentholated products suggests a relationship with menthol that should be further tested. Finally, we evaluated the bacterial communities of cigarette products stored under one environmental condition. Characterization of products stored under varying temperature and relative humidity conditions would enable us to better predict the impact of typical daily storage conditions (e.g., pocket conditions) on the dynamics of the bacterial communities in mentholated and non-mentholated cigarettes. Such experiments are currently ongoing in our laboratory. Even with these caveats, our study provides new knowledge regarding the bacterial constituents of commercially mentholated and non-mentholated tobacco products and the potential importance of these bacterial communities to human health.

From pre-harvest to puff, cigarette-associated bacteria are a culmination of ecosystems and commercial manipulations that result in a complex and diverse bacterial community, which may contribute to the acquisition and exchange of pathogenic and antibiotic resistance genes and/or species selection. Our data suggest that tobacco flavor additives, such as menthol, can affect the bacterial community composition of tobacco products and may lead to the selection or introduction of more resilient species. The bacteria and bacterial components present in non-mentholated and mentholated cigarettes may be introduced into the lung and oral cavity during the smoking process, carried by the filter-end of the cigarette butt and/or the tobacco particulate matter within mainstream smoke [21, 24, 26, 28]. These bacterial communities could play a direct role in the development of infectious and/or chronic illnesses among users or exacerbate existing negative health effects associated with smoking.

## Conclusions

This study comprehensively characterizes the complex bacterial communities residing in mentholated and non-mentholated cigarette products, which include bacterial pathogens of importance to public health. Most importantly, our study also shows that mentholation of cigarette products, a process used to reduce the harshness of cigarette products and appeal to a wider spectrum of consumers, significantly impacts the bacterial community of these products. Mentholation appeared to be correlated with a reduction in potential human bacterial pathogens and an increase in bacterial species resistant to harsh environmental conditions. These findings have critical implications regarding exposure to potentially infectious pathogens among cigarette smokers, and can be used to inform future tobacco control policies focused on the microbiology of tobacco, an understudied focus area in tobacco regulatory science.

## Additional files

Additional file 1: List of Supplementary Figures and Tables. **Figure S1.** Rarefaction curves for each product. **Figure S2.** PCoA analysis plots of weighted and unweighted Unifrac computed distances between cigarette products. **Figure S3.** Pseudomonas phylogenetic tree. **Figure S4.** Actinobacter phylogenetic tree. **Figure S5.** Stenotrophomonas phylogenetic tree. **Figure S6.** Anoxybacillus phylogenetic tree. **Figure S7.** Deinococcus phylogenetic tree. **Figure S8.** Vagococcus phylogenetic tree. **Figure S9.** Thermus phylogenetic tree. **Figure S10.** Proteus phylogenetic tree. **Table S1.** OTUs at statistically significantly different relative abundances between mentholated Camel King and non-mentholated Camel King. FC denotes fold change. (DOCX 1485 kb)

## Abbreviations

ANOSIM: Analysis of similarities
CC: Camel Crush
CCM: User-mentholated Camel Crush
CK: Camel Kings
CKM: Custom-mentholated Camel Kings
COPD: Chronic obstructive pulmonary disease
MAFFT: Multiple alignment using fast Fourier transform
NMB: Newport menthol box
OTU: Operational taxonomic unit
PBS: Phosphate-buffered saline
PC1: First principal component
PC2: Second principal component
QIIME: Quantitative insights into microbial ecology
RDP: Ribosomal database project
