# Supplementary material for: Mentholation affects the cigarette microbiota by selecting for bacteria resistant to harsh environmental conditions and selecting against potential bacterial pathogens
Source: Microbiome. 2017 Feb 15;5:22. doi: 10.1186/s40168-017-0235-0 (PMC5312438; doi:10.1186/s40168-017-0235-0)
Supplement: Additional file 1: — List of Supplementary Figures and Tables. Figure S1. Rarefaction curves for each product. Figure S2. PCoA analysis plots of weighted and unweighted Unifrac computed distances between cigarette products. Figure S3. Pseudomonas phylogenetic tree. Figure S4. Actinobacter phylogenetic tree. Figure S5. Stenotrophomonas phylogenetic tree. Figure S6. Anoxybacillus phylogenetic tree. Figure S7. Deinococcus phylogenetic tree. Figure S8. Vagococcus phylogenetic tree. Figure S9. Thermus phylogenetic tree. Figure S10. Proteus phylogenetic tree. Table S1. OTUs at statistically significantly different relative abundances between mentholated Camel King and non-mentholated Camel King. FC denotes fold change. (DOCX 1485 kb) [file 40168_2017_235_MOESM1_ESM.docx]

**Supplementary Figures and Tables**

**Mentholation affects the cigarette microbiota by selecting for bacteria resistant to harsh environmental conditions and selecting against potential bacterial pathogens**

Suhana Chattopadhyay^1,#^, Jessica Chopyk^1,#^, Prachi Kulkarni^1^, Emma Claye^1^, Kelsey R. Babik^1^, Molly C. Reid^1^, Eoghan M. Smyth^1,2^, Lauren E. Hittle^2^, Joseph N. Paulson^3^, Raul Cruz-Cano^4^, Mihai Pop^3^, Stephanie S. Buehler^5^, Pamela I. Clark^6^, Amy R. Sapkota^1,*^, Emmanuel F. Mongodin^2,*^

^1^ Maryland Institute for Applied Environmental Health, University of Maryland School of Public Health, College Park, MD, USA; ^2^ Institute for Genome Sciences and Department of Microbiology and Immunology, University of Maryland, School of Medicine, Baltimore, MD, USA; ^3^ Center for Bioinformatics and Computational Biology, University of Maryland, College Park, MD, USA; ^4^ Department of Epidemiology and Biostatistics, University of Maryland School of Public Health, College Park, MD, USA; ^5^ Public Health Center for Tobacco Research, Battelle, Columbus, OH, ^6^ Department of Behavioral and Community Health, University of Maryland School of Public Health, College Park, MD, USA

^#^ These authors contributed equally and are co-first authors.

* These authors contributed equally and are joint senior authors.

**Running title:** Bacterial communities in menthol and non-menthol cigarettes

Corresponding Author information:

Emmanuel F. Mongodin, PhD.

University of Maryland School of Medicine, Institute for Genome Sciences, 801 West Baltimore Street, Office #622, Baltimore, MD 21201, USA

**Figure S1:** Rarefaction curves for each product. Rarefaction curves for observed OTU counts for cigarette products CC, CCM, CK, CKM, and NMB. Each line represents a separate sample, with colors added to aid in visually discriminating samples.

**Figure S2:** PCoA analysis plots of weighted and unweighted Unifrac computed distances between cigarette products. (A) Points colored by brand: purple—Newport Menthol (NMB); green—mentholated Camel King (CKM); blue— mentholated Camel Crush (CCM); orange—Camel Kings (CK); red—Camel Crush (CC).

**Figure S3: *Pseudomonas* phylogenetic tree**. Unrooted maximum likelihood tree with 10 bootstrap replicates of representative sequences from the genera *Pseudomonas* and the OTUs of interest, *Pseudomonas putida* (OTU #3), *Pseudomonas oryzihabitans* (OTU #8, 1868), *Pseudomonas* sp. (OTU #10, 77, 132, 134, 163, 251, 608, 972, 1250, 1532, 1872, 1886), *Pseudomonas aeruginosa* (OTU #420), and *Pseudomonas fulva* (OTU #1137). Branch color denotes species level identification. Color key depicts the total number of strains of each species represented on the tree. The “Other” category consists of 11 strains of *Pseudomonas* *mandelii,* 11 *Pseudomonas* *libanensis,* 11 *Pseudomonas* *jessenii,* and 4 *Pseudomonas* *cichorii.*

**Figure S4: *Actinobacter*** **phylogenetic tree**. Unrooted maximum likelihood tree with 10 bootstrap replicates of representative sequences from the genera *Actinobacter* and the OTU of interest, *Actinobacter* sp. (OTU #12, 182, 247, 870, 1900), *Acinetobacter baumannii* (OTU #29), and *Acinetobacter calcoaceticus* (OTU #40, 496). Branch color denotes species level identification. Color key depicts the total number of strains of each species represented on the tree. The “Other” category consists 10 strains of *Acinetobacter ursingii* and *Acinetobacter* *gandensis*, 9 strains of *Acinetobacter nectaris*, and 8 strains of *Acinetobacter rhizosphaerae*.

**Figure S5: *Stenotrophomonas*** **phylogenetic tree**. Unrooted maximum likelihood tree with 10 bootstrap replicates of representative sequences from the genera *Stenotrophomonas* and the OTU of interest: *Stenotrophomonas* sp. (OTU #1682, 1899, 1913), and *Stenotrophomonas maltophilia* (OTU #15). Branch color denotes species level identification. Color key depicts the total number of strains of each species represented on the tree.

**Figure S6: *Anoxybacillus* phylogenetic tree**. Unrooted maximum likelihood tree with 10 bootstrap replicates of representative sequences from the genera *Anoxybacillus* and the OTU of interest, *Anoxybacillus* sp. OTU #31. Branch color denotes species level identification. Color key depicts the total number of strains of each species represented on the tree. The “Other” category consists of one strain each of *Anoxybacillus salavatliensis*, *Anoxybacillus hidirlerensis*, *Anoxybacillus eryuanensis*, *Anoxybacillus calidus*, *Anoxybacillus caldiproteolyticus*, *Anoxybacillus bogrovensis*, and *Anoxybacillus ayderensis*.

**Figure S7: *Deinococcus*** **phylogenetic tree**. Unrooted maximum likelihood tree with 10 bootstrap replicates of representative sequences from the genera *Deinococcus* and the OTU of interest, *Deinococcus* sp. OTU #272. Branch color denotes species level identification. Color key depicts the total number of strains of each species represented on the tree.

**Figure S8: *Vagococcus*** **phylogenetic tree**. Unrooted maximum likelihood tree with 10 bootstrap replicates of representative sequences from the genera *Vagococcus* and the OTU of interest, *Vagococcus* sp. OTU #54. Branch color denotes species level identification. Color key depicts the total number of strains of each species represented on the tree.

**Figure S9: *Thermus*** **phylogenetic tree**. Unrooted maximum likelihood tree with 10 bootstrap replicates of representative sequences from the genera *Thermus* and the OTU of interest, *Thermus* sp. OTU #266. Branch color denotes species level identification. Color key depicts the total number of strains of each species represented on the tree.

**Figure S10: *Proteus* phylogenetic tree**. Unrooted maximum likelihood tree with 10 bootstrap replicates of representative sequences from the genera *Proteus* and the OTU of interest, *Proteus* sp. OTU #450. Branch color denotes species level identification. Color key depicts the total number of strains of each species represented on the tree.

**Table S1:** OTUs at statistically significantly different relative abundances between mentholated Camel King and non-mentholated Camel King. FC denotes fold change.

| **OTU** | **log2 FC** | **pvalue** | **padj** | **Phylum** | **Genus** | **Gram** |
| --- | --- | --- | --- | --- | --- | --- |
| 2 | -1.56 | 1.18E-08 | 7.89E-07 | Proteobacteria | Sphingomonas sp. | Neg. |
| 3 | -1.77 | 1.33E-08 | 8.18E-07 | Proteobacteria | Pseudomonas putida | Neg. |
| 4 | -1.67 | 4.71E-06 | 8.74E-05 | Proteobacteria | Enterobacter sp. | Neg. |
| 5 | -1.68 | 4.63E-05 | 5.77E-04 | Firmicutes | Bacillus pumilus | Pos. |
| 6 | -1.72 | 7.65E-06 | 1.29E-04 | Firmicutes | Terribacillus sp. | Pos. |
| 7 | -1.52 | 3.72E-05 | 5.12E-04 | Firmicutes | Staphylococcus sp. | Pos. |
| 8 | -1.33 | 2.42E-04 | 2.10E-03 | Proteobacteria | Pseudomonas oryzihabitans | Neg. |
| 9 | -1.31 | 2.21E-03 | 1.36E-02 | Firmicutes | Bacillus clausii | Pos. |
| 11 | -1.82 | 1.40E-07 | 5.60E-06 | Proteobacteria | Rhizobium sp. | Neg. |
| 12 | -1.55 | 5.57E-04 | 4.28E-03 | Proteobacteria | Acinetobacter sp. | Neg. |
| 13 | -2.78 | 4.81E-11 | 1.09E-08 | Proteobacteria | Unknown Pseudomonadales | Neg. |
| 15 | -1.69 | 1.07E-04 | 1.12E-03 | Proteobacteria | Stenotrophomonas maltophilia | Neg. |
| 16 | -1.86 | 4.60E-05 | 5.77E-04 | Proteobacteria | Achromobacter sp. HJ-31-2 | Neg. |
| 17 | -1.71 | 4.26E-05 | 5.57E-04 | Firmicutes | Enterococcus sp. | Pos. |
| 18 | -1.30 | 5.86E-06 | 1.02E-04 | Proteobacteria | Methylobacterium sp. | Neg. |
| 19 | -1.32 | 2.85E-04 | 2.35E-03 | Firmicutes | Saccharibacillus sp. | Pos. |
| 23 | -1.18 | 3.64E-05 | 5.10E-04 | Proteobacteria | Unknown Aurantimonadaceae | Neg. |
| 24 | -1.42 | 2.18E-04 | 1.96E-03 | Proteobacteria | Paracoccus sp. | Neg. |
| 27 | -1.95 | 2.99E-07 | 1.08E-05 | Proteobacteria | Novosphingobium sp. | Neg. |
| 28 | -1.43 | 1.48E-06 | 4.12E-05 | Proteobacteria | Methylobacterium sp. | Neg. |
| 29 | -1.22 | 3.46E-03 | 2.01E-02 | Proteobacteria | Acinetobacter baumannii | Neg. |
| 30 | -1.64 | 1.41E-04 | 1.39E-03 | Firmicutes | Bacillus sp. | Pos. |
| 31 | 1.82 | 3.80E-06 | 7.77E-05 | Firmicutes | Anoxybacillus sp. | Pos. |
| 32 | -0.94 | 1.03E-02 | 4.74E-02 | Proteobacteria | Sphingomonas sp. | Neg. |
| 34 | -2.50 | 1.19E-08 | 7.89E-07 | Proteobacteria | Unknown Comamonadaceae | Neg. |
| 36 | -1.36 | 1.50E-06 | 4.12E-05 | Proteobacteria | Methylobacterium sp. | Neg. |
| 37 | -2.83 | 5.47E-11 | 1.09E-08 | Firmicutes | Paenibacillus amylolyticus | Pos. |
| 39 | -1.41 | 1.29E-03 | 8.50E-03 | Proteobacteria | Achromobacter sp. | Neg. |
| 40 | -1.81 | 6.21E-05 | 7.08E-04 | Proteobacteria | Acinetobacter calcoaceticus | Neg. |
| 41 | -1.10 | 4.35E-03 | 2.36E-02 | Firmicutes | Bacillus licheniformis | Pos. |
| 44 | -2.60 | 1.78E-09 | 2.36E-07 | Proteobacteria | Luteimonas sp. | Neg. |
| 46 | -1.55 | 2.74E-04 | 2.28E-03 | Proteobacteria | Ochrobactrum sp. | Neg. |
| 49 | -2.35 | 1.81E-08 | 9.63E-07 | Proteobacteria | Achromobacter sp. | Neg. |
| 51 | -2.18 | 1.55E-06 | 4.13E-05 | Firmicutes | Paenibacillus sp. icri4 | Pos. |
| 52 | -1.70 | 1.65E-04 | 1.55E-03 | Firmicutes | Streptomyces sp. KP17 | Pos. |
| 54 | 1.10 | 7.44E-03 | 3.71E-02 | Firmicutes | Vagococcus sp. | Pos. |
| 55 | -1.43 | 3.32E-04 | 2.68E-03 | Actinobacteria | Brachybacterium sp. | Pos. |
| 56 | -2.11 | 8.94E-07 | 2.64E-05 | Proteobacteria | Brevundimonas sp. | Neg. |
| 59 | -1.77 | 3.88E-05 | 5.16E-04 | Bacteroidetes | Sphingobacterium sp. | Neg. |
| 61 | -2.80 | 1.74E-10 | 2.77E-08 | Bacteroidetes | Sphingobacterium sp. | Neg. |
| 65 | -1.30 | 2.97E-04 | 2.42E-03 | Actinobacteria | Curtobacterium sp. | Pos. |
| 67 | -1.23 | 1.77E-03 | 1.14E-02 | Firmicutes | Unknown Bacillaceae | Pos. |
| 74 | -1.81 | 8.31E-06 | 1.35E-04 | Proteobacteria | Paracoccus sp. | Neg. |
| 75 | -1.30 | 1.22E-03 | 8.08E-03 | Firmicutes | Paenibacillus sp. | Pos. |
| 77 | -0.97 | 8.68E-03 | 4.20E-02 | Proteobacteria | Pseudomonas sp. | Neg. |
| 86 | -1.02 | 1.06E-02 | 4.84E-02 | Actinobacteria | Nocardioides sp. | Pos. |
| 87 | 0.94 | 1.03E-02 | 4.74E-02 | Proteobacteria | Schlegelella sp. | Neg. |
| 88 | -2.01 | 2.28E-06 | 5.21E-05 | Bacteroidetes | Sphingobacterium sp. | Neg. |
| 90 | -1.48 | 1.48E-04 | 1.44E-03 | Firmicutes | Solibacillus sp. | Pos. |
| 91 | -2.50 | 5.57E-09 | 5.12E-07 | Firmicutes | Paenibacillus montaniterrae | Pos. |
| 93 | -1.11 | 9.34E-03 | 4.37E-02 | Firmicutes | Lysinibacillus sp. | Pos. |
| 95 | -1.54 | 1.16E-04 | 1.19E-03 | Actinobacteria | Unknown Cellulomonadaceae | Pos. |
| 97 | -1.93 | 3.20E-07 | 1.11E-05 | Firmicutes | Unknown Lachnospiraceae | Pos. |
| 98 | -1.54 | 2.61E-04 | 2.19E-03 | Firmicutes | Oceanobacillus sp. | Pos. |
| 101 | -1.58 | 4.92E-06 | 8.93E-05 | Proteobacteria | Methylobacterium sp. | Neg. |
| 103 | -3.12 | 1.30E-14 | 1.04E-11 | Proteobacteria | Brevundimonas sp. | Neg. |
| 110 | -1.39 | 8.02E-04 | 5.93E-03 | Proteobacteria | Ochrobactrum sp. | Neg. |
| 111 | -1.18 | 3.48E-03 | 2.01E-02 | Proteobacteria | Cellvibrio sp. | Neg. |
| 112 | -1.08 | 6.53E-03 | 3.34E-02 | Proteobacteria | Roseomonas sp. | Neg. |
| 116 | -1.66 | 2.89E-06 | 6.24E-05 | Proteobacteria | Unknown Aurantimonadaceae | Neg. |
| 122 | -1.36 | 3.80E-04 | 3.00E-03 | Firmicutes | Bacillus sp. | Pos. |
| 125 | -2.31 | 7.72E-08 | 3.24E-06 | Proteobacteria | Achromobacter sp. | Neg. |
| 127 | -1.34 | 1.17E-03 | 7.89E-03 | Proteobacteria | Achromobacter sp. | Neg. |
| 132 | -1.93 | 9.26E-06 | 1.48E-04 | Proteobacteria | Pseudomonas sp. | Neg. |
| 135 | -1.43 | 1.38E-04 | 1.38E-03 | Firmicutes | Marinilactibacillus sp. | Pos. |
| 143 | -2.08 | 5.89E-07 | 1.81E-05 | Firmicutes | Staphylococcus sp. | Pos. |
| 146 | -1.57 | 1.13E-05 | 1.77E-04 | Firmicutes | Natribacillus sp. | Pos. |
| 148 | -1.44 | 1.59E-04 | 1.51E-03 | Proteobacteria | Shigella sp. | Neg. |
| 152 | -1.43 | 1.94E-04 | 1.80E-03 | Firmicutes | Unknown Planococcaceae | Pos. |
| 156 | -1.12 | 6.48E-03 | 3.34E-02 | Bacteroidetes | Sphingobacterium sp. | Neg. |
| 159 | -1.47 | 2.47E-05 | 3.65E-04 | Proteobacteria | Methylobacterium sp. | Neg. |
| 163 | -1.42 | 2.60E-04 | 2.19E-03 | Proteobacteria | Pseudomonas sp. | Neg. |
| 166 | -1.15 | 3.57E-03 | 2.05E-02 | Firmicutes | Paenibacillus sp. | Pos. |
| 167 | -1.83 | 2.00E-06 | 4.83E-05 | Proteobacteria | Azospirillum irakense | Neg. |
| 176 | -1.60 | 4.33E-05 | 5.57E-04 | Firmicutes | Bacillus cereus | Pos. |
| 179 | -1.32 | 1.03E-03 | 7.20E-03 | Actinobacteria | Cellulosimicrobium sp. | Pos. |
| 182 | -1.28 | 3.56E-04 | 2.84E-03 | Proteobacteria | Acinetobacter sp. | Neg. |
| 189 | -1.07 | 2.87E-03 | 1.71E-02 | Firmicutes | Bacillus sp. | Pos. |
| 193 | -1.51 | 5.38E-05 | 6.60E-04 | Actinobacteria | Unknown Bogoriellaceae | Pos. |
| 195 | -1.08 | 5.83E-03 | 3.10E-02 | Proteobacteria | Sphingobium sp. | Neg. |
| 196 | -1.05 | 8.55E-03 | 4.18E-02 | Firmicutes | Paenibacillus sp. | Pos. |
| 200 | -2.45 | 6.79E-09 | 5.42E-07 | Proteobacteria | Unknown Alcaligenaceae | Neg. |
| 202 | -1.50 | 5.58E-05 | 6.75E-04 | Proteobacteria | Tatumella sp. | Neg. |
| 206 | -1.15 | 8.84E-03 | 4.25E-02 | Firmicutes | Oceanobacillus sp. | Pos. |
| 207 | 0.93 | 9.05E-03 | 4.33E-02 | Proteobacteria | Silanimonas sp. | Neg. |
| 210 | -1.39 | 1.98E-04 | 1.82E-03 | Proteobacteria | Methylobacillus sp. | Neg. |
| 211 | -1.07 | 2.08E-03 | 1.30E-02 | Actinobacteria | Unknown Beutenbergiaceae | Pos. |
| 212 | -1.41 | 1.16E-04 | 1.19E-03 | Proteobacteria | Devosia sp. | Neg. |
| 214 | -2.21 | 4.06E-09 | 4.63E-07 | Firmicutes | Brevibacillus sp. | Pos. |
| 215 | -1.00 | 6.90E-03 | 3.51E-02 | Firmicutes | Bacillus sp. | Pos. |
| 217 | -1.03 | 6.94E-03 | 3.51E-02 | Firmicutes | Bacillus sp. | Pos. |
| 225 | -1.29 | 1.53E-03 | 9.95E-03 | Proteobacteria | Brevundimonas sp. | Neg. |
| 247 | -0.99 | 9.92E-03 | 4.60E-02 | Proteobacteria | Acinetobacter sp. | Neg. |
| 251 | -1.10 | 4.31E-03 | 2.36E-02 | Proteobacteria | Pseudomonas sp. | Neg. |
| 254 | -0.98 | 3.72E-03 | 2.12E-02 | Firmicutes | Ureibacillus sp. | Pos. |
| 256 | -1.41 | 7.67E-04 | 5.72E-03 | Proteobacteria | Unknown Enterobacteriaceae | Neg. |
| 257 | -1.50 | 8.07E-05 | 8.95E-04 | Firmicutes | Paenibacillus sp. | Pos. |
| 262 | -1.07 | 5.60E-03 | 3.00E-02 | Proteobacteria | Luteimonas sp. | Neg. |
| 266 | 1.01 | 6.06E-03 | 3.17E-02 | Deinococcus-Thermus | Thermus sp. | Pos. |
| 267 | -1.08 | 2.43E-03 | 1.48E-02 | Bacteroidetes | Flavobacterium sp. | Neg. |
| 272 | 1.03 | 4.27E-03 | 2.36E-02 | Deinococcus-Thermus | Deinococcus | Pos. |
| 280 | -1.23 | 9.15E-04 | 6.70E-03 | Proteobacteria | Asticcacaulis sp. | Neg. |
| 285 | -1.00 | 9.37E-03 | 4.37E-02 | Proteobacteria | Aeromonas sp. | Neg. |
| 321 | -1.07 | 2.25E-03 | 1.38E-02 | Bacteroidetes | Flavobacterium sp. | Neg. |
| 326 | -1.10 | 1.79E-03 | 1.14E-02 | Bacteroidetes | Myroides sp. | Neg. |
| 340 | -1.12 | 2.13E-03 | 1.33E-02 | Firmicutes | Paenibacillus sp. | Pos. |
| 364 | -1.81 | 4.11E-06 | 8.00E-05 | Proteobacteria | Ochrobactrum sp. | Neg. |
| 375 | -0.97 | 6.07E-03 | 3.17E-02 | Proteobacteria | Sphingomonas sp. | Neg. |
| 390 | -1.18 | 9.26E-04 | 6.71E-03 | Proteobacteria | Lampropedia sp. | Neg. |
| 398 | -1.92 | 5.78E-09 | 5.12E-07 | Proteobacteria | Pantoea sp. | Neg. |
| 401 | -1.57 | 2.52E-05 | 3.65E-04 | Firmicutes | Lysinibacillus sp. | Pos. |
| 415 | -1.17 | 3.97E-03 | 2.23E-02 | Bacteroidetes | Chryseobacterium sp. | Neg. |
| 420 | -1.34 | 2.53E-03 | 1.53E-02 | Proteobacteria | Pseudomonas aeruginosa | Neg. |
| 425 | -1.88 | 1.56E-05 | 2.39E-04 | Proteobacteria | Rheinheimera sp. | Neg. |
| 446 | -1.30 | 2.31E-04 | 2.05E-03 | Proteobacteria | Erwinia chrysanthemi | Neg. |
| 450 | -1.24 | 4.60E-04 | 3.56E-03 | Proteobacteria | Proteus mirabilis | Neg. |
| 496 | -2.14 | 2.35E-06 | 5.21E-05 | Proteobacteria | Acinetobacter calcoaceticus | Neg. |
| 502 | -1.19 | 1.18E-03 | 7.94E-03 | Proteobacteria | Methylobacterium sp. | Neg. |
| 530 | -1.47 | 2.12E-05 | 3.19E-04 | Firmicutes | Bacillus novalis | Pos. |
| 537 | -1.03 | 8.13E-03 | 4.03E-02 | Proteobacteria | Unknown Enterobacteriaceae | Neg. |
| 578 | -1.01 | 4.35E-03 | 2.36E-02 | Bacteroidetes | Fluviicola sp. | Neg. |
| 608 | -0.94 | 8.35E-03 | 4.12E-02 | Proteobacteria | Pseudomonas sp. | Neg. |
| 667 | -2.06 | 4.32E-07 | 1.44E-05 | Firmicutes | Unknown Bacillales | Pos. |
| 692 | -1.01 | 7.25E-03 | 3.64E-02 | Proteobacteria | Sphingopyxis sp. | Neg. |
| 725 | -1.05 | 5.98E-03 | 3.16E-02 | Proteobacteria | Pantoea sp. | Neg. |
| 757 | -2.19 | 3.64E-08 | 1.61E-06 | Proteobacteria | Rhizobium sp. | Neg. |
| 972 | -1.20 | 5.73E-04 | 4.35E-03 | Proteobacteria | Pseudomonas sp. | Neg. |
| 1128 | -1.46 | 6.00E-05 | 7.06E-04 | Bacteroidetes | Chryseobacterium sp. | Neg. |
| 1192 | -1.17 | 5.01E-03 | 2.70E-02 | Proteobacteria | Unknown Rhodobacteraceae | Neg. |
| 1239 | -2.01 | 1.83E-06 | 4.58E-05 | Proteobacteria | Unknown Enterobacteriaceae | Neg. |
| 1247 | -1.57 | 4.52E-06 | 8.59E-05 | Actinobacteria | Leucobacter sp. | Pos. |
| 1270 | -1.84 | 2.92E-05 | 4.16E-04 | Proteobacteria | Enterobacter sp. | Neg. |
| 1287 | -1.22 | 4.58E-04 | 3.56E-03 | Proteobacteria | Sphingomonas sp. | Neg. |
| 1442 | -1.54 | 7.79E-06 | 1.29E-04 | Firmicutes | Bacillus novalis | Pos. |
| 1448 | -1.72 | 1.68E-06 | 4.32E-05 | Proteobacteria | Pantoea sp. | Neg. |
| 1513 | -1.57 | 3.87E-05 | 5.16E-04 | Proteobacteria | Enterobacter sp. | Neg. |
| 1532 | -1.48 | 7.35E-04 | 5.53E-03 | Proteobacteria | Pseudomonas sp. | Neg. |
| 1609 | -1.06 | 9.27E-03 | 4.37E-02 | Proteobacteria | Achromobacter sp. | Neg. |
| 1621 | -0.94 | 4.21E-03 | 2.35E-02 | Proteobacteria | Sphingomonas sp. | Neg. |
| 1671 | -1.54 | 1.37E-04 | 1.38E-03 | Proteobacteria | Massilia sp. | Neg. |
| 1681 | -1.56 | 1.52E-04 | 1.47E-03 | Firmicutes | Saccharibacillus sp. | Pos. |
| 1682 | -1.58 | 6.02E-05 | 7.06E-04 | Proteobacteria | Stenotrophomonas sp. | Neg. |
| 1730 | -1.09 | 2.79E-03 | 1.67E-02 | Proteobacteria | Massilia sp. | Neg. |
| 1797 | -0.93 | 9.30E-03 | 4.37E-02 | Proteobacteria | Sphingomonas sp. | Neg. |
| 1805 | -1.40 | 1.45E-03 | 9.50E-03 | Proteobacteria | Achromobacter sp. | Neg. |
| 1817 | -1.29 | 1.14E-03 | 7.79E-03 | Bacteroidetes | Sphingobacterium sp. | Neg. |
| 1835 | -1.02 | 3.19E-03 | 1.89E-02 | Proteobacteria | Klebsiella sp | Neg. |
| 1850 | -1.20 | 6.87E-05 | 7.72E-04 | Proteobacteria | Sphingomonas sp. | Neg. |
| 1868 | -1.05 | 9.72E-04 | 6.92E-03 | Proteobacteria | Pseudomonas oryzihabitans | Neg. |
| 1872 | -1.49 | 9.26E-05 | 1.01E-03 | Proteobacteria | Pseudomonas sp. | Neg. |
| 1879 | -1.22 | 1.08E-03 | 7.45E-03 | Proteobacteria | Pantoea sp. | Neg. |
| 1885 | -1.73 | 5.77E-07 | 1.81E-05 | Proteobacteria | Unknown Enterobacteriaceae | Neg. |
| 1899 | -1.16 | 1.03E-03 | 7.20E-03 | Proteobacteria | Stenotrophomonas sp. | Neg. |
| 1900 | -1.63 | 2.35E-04 | 2.06E-03 | Proteobacteria | Acinetobacter sp. | Neg. |
| 1904 | -1.18 | 1.07E-03 | 7.42E-03 | Proteobacteria | Pantoea sp. | Neg. |
| 1913 | -2.46 | 2.88E-08 | 1.35E-06 | Proteobacteria | Stenotrophomonas sp. | Neg. |
| 1915 | -0.96 | 6.37E-03 | 3.30E-02 | Proteobacteria | Klebsiella sp | Neg. |
| 1937 | -2.36 | 9.76E-12 | 3.90E-09 | Firmicutes | Bacillus pumilus | Pos. |
| 1948 | -1.86 | 3.36E-06 | 7.05E-05 | Firmicutes | Bacillus pumilus | Pos. |
| 1951 | -2.08 | 2.03E-08 | 1.01E-06 | Firmicutes | Unknown Bacillaceae | Pos. |
| 1956 | -1.49 | 9.52E-04 | 6.85E-03 | Proteobacteria | Unknown Alcaligenaceae | Neg. |
| 1969 | -1.71 | 1.61E-08 | 9.20E-07 | Proteobacteria | Unknown Enterobacteriaceae | Neg. |
| 1983 | -1.85 | 2.34E-06 | 5.21E-05 | Proteobacteria | Unknown Enterobacteriaceae | Neg. |
| 1987 | -1.00 | 8.58E-03 | 4.18E-02 | Proteobacteria | Falsirhodobacter sp. | Neg. |
| 1998 | -1.21 | 3.90E-03 | 2.21E-02 | Proteobacteria | Agrobacterium tumefaciens | Neg. |
| 2017 | -1.41 | 2.59E-04 | 2.19E-03 | Proteobacteria | Unknown Enterobacteriaceae | Neg. |
| 2018 | -2.07 | 4.09E-06 | 8.00E-05 | Proteobacteria | Unknown Enterobacteriaceae | Neg. |
| 2038 | -1.19 | 2.04E-03 | 1.29E-02 | Proteobacteria | Unknown Alcaligenaceae | Neg. |
| 2039 | -1.47 | 6.13E-05 | 7.08E-04 | Proteobacteria | Pantoea sp. | Neg. |
| 2045 | -1.34 | 2.19E-04 | 1.96E-03 | Proteobacteria | Pantoea sp. | Neg. |
| 2046 | -1.33 | 9.69E-05 | 1.03E-03 | Proteobacteria | Unknown Enterobacteriaceae | Neg. |
| 2047 | -1.61 | 9.36E-05 | 1.01E-03 | Firmicutes | Bacillus sp. | Pos. |
| 2052 | -1.25 | 3.38E-03 | 1.98E-02 | Proteobacteria | Massilia sp. | Neg. |

**Table S2:** OTUs at statistically significantly different relative abundances between mentholated Camel Crush and non-mentholated Camel Crush. FC denotes fold change.

| **OTU** | **log2 FC** | **pvalue** | **padj** | **Phylum** | **Genus** | **Gram** |
| --- | --- | --- | --- | --- | --- | --- |
| 667 | -3.19 | 5.78E-15 | 4.04E-12 | Firmicutes | Unknown Bacillales | Pos. |
| 30 | -2.91 | 2.39E-11 | 8.35E-09 | Firmicutes | Bacillus sp. | Pos. |
| 104 | -1.96 | 1.46E-06 | 1.45E-04 | Firmicutes | Facklamia sp. | Pos. |
| 73 | -1.87 | 1.25E-05 | 8.74E-04 | Firmicutes | Jeotgalicoccus sp. | Pos. |
| 285 | -1.85 | 7.41E-07 | 8.62E-05 | Proteobacteria | Aeromonas sp. | Neg. |
| 143 | -1.59 | 1.64E-04 | 4.10E-03 | Firmicutes | Staphylococcus sp. | Pos. |
| 783 | -1.56 | 2.58E-05 | 1.38E-03 | Proteobacteria | Cedecea sp. | Neg. |
| 333 | -1.53 | 2.76E-05 | 1.38E-03 | Proteobacteria | Unknown Sphingomonadales | Neg. |
| 128 | -1.46 | 9.66E-05 | 2.86E-03 | Firmicutes | Paenibacillus sp. | Pos. |
| 2017 | -1.44 | 9.83E-05 | 2.86E-03 | Proteobacteria | Unknown Enterobacteriaceae | Neg. |
| 293 | -1.37 | 1.06E-04 | 2.96E-03 | Actinobacteria | Saccharopolyspora sp. | Pos. |
| 1729 | -1.29 | 1.01E-03 | 1.76E-02 | Actinobacteria | Unknown Streptomycetaceae | Pos. |
| 86 | -1.25 | 1.73E-03 | 2.57E-02 | Actinobacteria | Nocardioides sp. | Pos. |
| 1448 | -1.21 | 8.11E-04 | 1.62E-02 | Proteobacteria | Pantoea sp. | Neg. |
| 124 | -1.20 | 1.51E-03 | 2.34E-02 | Bacteroidetes | Sphingobacterium sp. | Neg. |
| 340 | -1.15 | 1.89E-03 | 2.75E-02 | Firmicutes | Paenibacillus sp. | Pos. |
| 1682 | -1.13 | 3.68E-03 | 4.43E-02 | Proteobacteria | Stenotrophomonas sp. | Neg. |
| 289 | -1.09 | 2.88E-03 | 3.65E-02 | Proteobacteria | Paracoccus sp. | Neg. |
| 296 | -1.08 | 2.25E-03 | 2.99E-02 | Firmicutes | Unknown Bacillaceae | Pos. |
| 193 | -1.07 | 4.03E-03 | 4.74E-02 | Actinobacteria | Unknown Bogoriellaceae | Pos. |
| 398 | -1.01 | 2.64E-03 | 3.42E-02 | Proteobacteria | Pantoea sp. | Neg. |
| 1969 | -0.96 | 1.96E-03 | 2.75E-02 | Proteobacteria | Unknown Enterobacteriaceae | Neg. |
| 535 | 1.04 | 4.07E-03 | 4.74E-02 | Actinobacteria | Bifidobacterium sp. | Pos. |
| 359 | 1.13 | 2.27E-03 | 2.99E-02 | Firmicutes | Lactobacillus plantarum | Pos. |
| 220 | 1.14 | 1.08E-03 | 1.84E-02 | Proteobacteria | Tepidimonas sp. | Neg. |
| 272 | 1.20 | 9.64E-04 | 1.76E-02 | Deinococcus-Thermus | Deinococcus sp. | Pos. |
| 300 | 1.22 | 1.28E-03 | 2.10E-02 | Proteobacteria | Cupriavidus sp. | Neg. |
| 153 | 1.23 | 1.97E-03 | 2.75E-02 | Actinobacteria | Brevibacterium sp. | Pos. |
| 112 | 1.27 | 1.55E-03 | 2.35E-02 | Proteobacteria | Roseomonas sp. | Neg. |
| 1182 | 1.29 | 1.32E-03 | 2.10E-02 | Firmicutes | Unknown Bacillales | Pos. |
| 1532 | 1.31 | 3.12E-03 | 3.83E-02 | Proteobacteria | Pseudomonas sp. | Neg. |
| 893 | 1.33 | 9.98E-04 | 1.76E-02 | Proteobacteria | Acidovorax sp. | Neg. |
| 14 | 1.33 | 3.10E-03 | 3.83E-02 | Firmicutes | Aerococcus sp. | Pos. |
| 163 | 1.35 | 5.56E-04 | 1.14E-02 | Proteobacteria | Pseudomonas sp. | Neg. |
| 20 | 1.36 | 1.29E-04 | 3.47E-03 | Proteobacteria | Achromobacter sp. | Neg. |
| 21 | 1.36 | 2.05E-03 | 2.80E-02 | Actinobacteria | Corynebacterium sp. | Pos. |
| 197 | 1.37 | 9.01E-04 | 1.75E-02 | Bacteroidetes | Pedobacter sp. | Neg. |
| 870 | 1.38 | 4.39E-04 | 9.37E-03 | Proteobacteria | Acinetobacter sp. | Neg. |
| 161 | 1.38 | 4.43E-04 | 9.37E-03 | Bacteroidetes | Sphingobacterium sp. | Neg. |
| 551 | 1.43 | 1.01E-03 | 1.76E-02 | Actinobacteria | Corynebacterium sp. | Pos. |
| 48 | 1.43 | 1.29E-03 | 2.10E-02 | Proteobacteria | Pectobacterium carotovorum | Neg. |
| 77 | 1.47 | 1.50E-04 | 3.89E-03 | Proteobacteria | Pseudomonas sp. | Neg. |
| 31 | 1.54 | 1.86E-04 | 4.46E-03 | Firmicutes | Anoxybacillus sp. | Pos. |
| 1900 | 1.56 | 4.25E-04 | 9.37E-03 | Proteobacteria | Acinetobacter sp. | Neg. |
| 331 | 1.57 | 2.15E-05 | 1.25E-03 | Bacteroidetes | Dysgonomonas sp. | Neg. |
| 126 | 1.58 | 4.55E-05 | 1.56E-03 | Bacteroidetes | Myroides sp. | Neg. |
| 88 | 1.61 | 1.92E-04 | 4.46E-03 | Bacteroidetes | Sphingobacterium sp. | Neg. |
| 253 | 1.63 | 1.17E-05 | 8.74E-04 | Proteobacteria | Pantoea sp. | Neg. |
| 90 | 1.66 | 3.54E-05 | 1.50E-03 | Firmicutes | Solibacillus sp. | Pos. |
| 204 | 1.67 | 4.04E-05 | 1.56E-03 | Bacteroidetes | Flavobacterium sp. | Neg. |
| 437 | 1.68 | 2.99E-06 | 2.61E-04 | Bacteroidetes | Proteiniphilum sp. | Neg. |
| 242 | 1.68 | 1.73E-05 | 1.10E-03 | Bacteroidetes | Epilithonimonas sp. | Neg. |
| 93 | 1.71 | 8.04E-05 | 2.55E-03 | Firmicutes | Lysinibacillus sp. | Pos. |
